# Malignant hyperthermia mutations and correlation with the severity of the anesthetic complication and the level of the in vitro contracture tests

**DOI:** 10.1186/1471-2253-14-S1-A1

**Published:** 2014-08-18

**Authors:** René Krivosic-Horber, Anne-Frédérique Dalmas, Etienne Brasdefer, Nicole Monnier, Valérie Deken

**Affiliations:** 1 Malignant Hyperthermia Unit, Academic Unit Anaesthesia, University Hospital Lille, Lille, 59037, France; 2 Laboratory of Molecular Biology, University Hospital Grenoble, Grenoble, 38043, France; 3 Laboratoire de biostatistiques, CHRU de Lille, Lille, 59037, France

## Background

Malignant hyperthermia (MH) is a complication of anaesthesia appearing as an acute potentially lethal hypermetabolic state in people carrying a genetic anomaly expressed in the skeletal muscle. MH susceptibility can be diagnosed by *in vitro* contracture test (IVCT) with halothane and caffeine requiring muscular biopsy, or by looking for the MH mutations directly in DNA extracted from the blood. Studies showed an influence of the type of mutation (genotype) on the various expressions (phenotypes) of the MH susceptibility. The aims of this study are to look for any correlation between the presence of a MH mutation and 1) the severity of the anesthetic complication and 2) the force of the contractures observed in the IVCT.

## Materials and methods

Observational analytical retrospective anonymized study based on the informations contained in the Lille MH Database. The criteria of inclusion were: 1) anesthetic probands classified as MHS according to the IVCT and/or the presence of a MH mutation in the RYR1 gene in at least one family member and 2) existence of sufficient clinical information concerning the crisis. The severity of the MH crisis was classified as: death, survival after stay in intensive care unit ICU, survival without stay in ICU. The results of the IVCT: maximal force of contracture at halothane 2%, caffeine 2mmol/L, halothane 3%. The statistical analysis was carried out with a test of Chi-2 for the comparisons of frequency and a test of Mann Whitney for the nonparametric variables with p<0,05 significant.

## Results

92 MHS probands were included in the study: 63 men (68%) and 29 women (32%) over a period going from 1964 to 2013. The age at the moment of the crisis is 18,9 years (DS 13,3). Mutations were found in 59 families (64%) including 53 causal mutations (58%) and 6 probable (6%). No significant difference was observed in the degree of severity between the 2 populations. The force of contracture of the IVCT was significantly higher in the group with MH (Table 1).

**Table 1:** Severity of malignant hyperthermia reaction and force of contracture of the In-vitro-contracture test

	N=92	No mutation	Mutation	p
**Severity**		n=33	n=59	
death	29	10 (35%)	19 (65%)	0,85*(NS)
Alive stay ICU	28	5 (18%)	23 (82%)	0,15*(NS)
Alive no stay ICU	35	18 (52%)	17 (48%)	0,17*(NS)

**Force of contracture**		**Mean+/-SD**	**Mean+/-SD**	

H2% g		0,99+/-1,02	2,46+/-1,86	0,00005**
Caf 2mmol g		0,58+/-0,58	1,29+/-0,97	0,00002**
H3% g		1,43+/-1,33	4,2+/-2,43	0,0000003**

## Conclusions

In this study, the severity of the MH crisis is not related to the presence of MH mutations. It is well known that the severity is more dependent on environmental factors, age of the crisis, exposure time to the volatile anesthetic agents, administration of dantrolene. On the other hand, the presence of a MH mutation in gene RYR1 modifies the muscular phenotype since the force of contracture in the IVCT is significantly more important in this group. These results support the genetic heterogeneity of the disease.

